# Spatially explicit multi-criteria decision analysis for managing vector-borne diseases

**DOI:** 10.1186/1476-072X-10-70

**Published:** 2011-12-29

**Authors:** Valerie Hongoh, Anne Gatewood Hoen, Cécile Aenishaenslin, Jean-Philippe Waaub, Denise Bélanger, Pascal Michel

**Affiliations:** 1 Groupe de Recherche en Épidémiologie des Zoonoses et Santé Publique (GREZOSP), Pavillon de la santé publique, Faculté de médecine vétérinaire, Université de Montréal, Case postale 5000, Saint-Hyacinthe, Québec, J2S 7C6, Canada; 2 Department of Community and Family Medicine, Dartmouth Medical School, HB 7937, One Medical Center Drive, Dartmouth-Hitchcock Medical Center, Lebanon, New Hampshire, 03756, USA; 3 Département de Géographie, Université du Québec à Montréal, Case postale 8888, Succursale Centre-ville, Montréal, Québec, H3C 3P8, Canada; 4 Laboratory for Foodborne Zoonoses, Public Health Agency of Canada, CP 5000, St-Hyacinthe, Québec, H2S 7C6, Canada

**Keywords:** spatial multi-criteria decision analysis, vector-borne disease, risk modeling

## Abstract

The complex epidemiology of vector-borne diseases creates significant challenges in the design and delivery of prevention and control strategies, especially in light of rapid social and environmental changes. Spatial models for predicting disease risk based on environmental factors such as climate and landscape have been developed for a number of important vector-borne diseases. The resulting risk maps have proven value for highlighting areas for targeting public health programs. However, these methods generally only offer technical information on the spatial distribution of disease risk itself, which may be incomplete for making decisions in a complex situation. In prioritizing surveillance and intervention strategies, decision-makers often also need to consider spatially explicit information on other important dimensions, such as the regional specificity of public acceptance, population vulnerability, resource availability, intervention effectiveness, and land use. There is a need for a unified strategy for supporting public health decision making that integrates available data for assessing spatially explicit disease risk, with other criteria, to implement effective prevention and control strategies. Multi-criteria decision analysis (MCDA) is a decision support tool that allows for the consideration of diverse quantitative and qualitative criteria using both data-driven and qualitative indicators for evaluating alternative strategies with transparency and stakeholder participation. Here we propose a MCDA-based approach to the development of geospatial models and spatially explicit decision support tools for the management of vector-borne diseases. We describe the conceptual framework that MCDA offers as well as technical considerations, approaches to implementation and expected outcomes. We conclude that MCDA is a powerful tool that offers tremendous potential for use in public health decision-making in general and vector-borne disease management in particular.

## Background

Many diseases are spatially constrained; for example, vector-borne and zoonotic diseases occur where and when vectors, animal hosts, pathogens and susceptible human populations overlap [1]. Vectors, pathogens and human populations are unevenly distributed in space and time and as a result risk for exposure to vector-borne diseases is spatially heterogeneous. Spatial models for the study and management of vector-borne disease risk have become common with the development of digitally encoded environmental data and computational tools such as geographical information systems (GIS). These are often presented as maps of expected distribution of vector abundance or risk for exposure to a pathogen, and can be created using a variety of statistical and algorithmic techniques many of which have been recently reviewed [2-7]. In addition to providing a geographical representation of risk, such models often help identify the underlying factors contributing to vector-borne disease risk and burden.

Risk maps have proven to be important tools for public health decision making and priority-setting for vector-borne diseases because they assist with the targeting of prevention and control efforts. However, public health decision-making routinely requires the consideration of complex factors beyond the geographical distribution and determinants of disease risk. Considerations related to individual and societal costs, perceived risk, strategic or policy-driven objectives, and resource allocation priorities are often necessary elements to be considered when designing public health actions, adding layers of complexity to an already complicated decision-making process. In addition, available spatial risk models for vector-borne disease generally characterize entomological risk, or risk of exposure to an infected disease vector, without incorporating data on other spatially-varying components such as the underlying distribution of vulnerable human populations. As decision support tools for vector-borne disease prevention and control, therefore, risk models are but one part of a whole. Finally, spatial risk models are only as good as the data from which they were created [4]; models based on incomplete, biased or sub-optimal data may still provide insight, but their inaccuracies and limitations must be taken under consideration.

**Figure 1:**
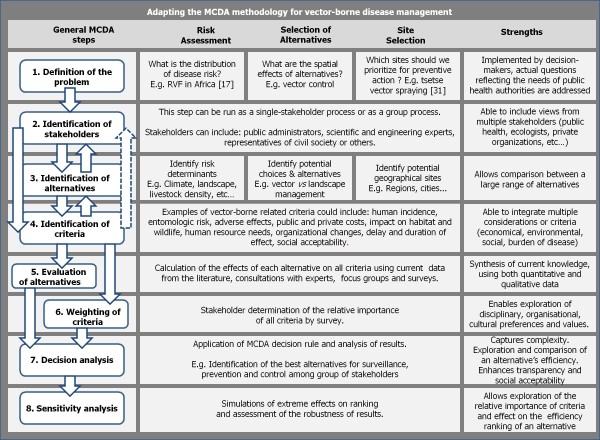
**General steps in an MCDA process adapted to risk assessment, selection of alternatives and site selection**. The steps in a general MCDA and spatial MCDA are similar. First, the objective of the analysis is defined (step1). Next, the key stakeholders that should be involved in the analysis process are identified (step 2). The following steps involve defining all possible alternatives under consideration (step 3) and all of the relevant criteria for evaluating these alternatives (step 4). These steps are interchangeable and may lead to an iterative process of refining which stakeholders to involve. Next, the alternatives are assessed based on the identified criteria (step 5). Performance indicators or decision variables are created for each intersecting pair of alternative and criteria. For spatial MCDA, evaluation criterion maps are generated to evaluate the performance of alternatives. Constraint maps can also be generated to display the limitations of the values that decision variables may assume. Following this, all criteria are weighted by participating stakeholders in order to reflect the preference values of those involved (step 6). It should be noted that not all MCDA approaches make use of weighting; other ordering techniques such as pair-wise comparison can be used. Next, a mathematical combination of the criteria is performed using a decision rule and effectively combines the results of the preceding four steps (step 7). The combined criteria produce an ordering of alternatives. Finally, a sensitivity analysis is performed to examine the robustness of the ranking outcome (step 8). The end result of the MCDA process is a recommendation consisting either of the best-ranked alternative or group of alternatives.

The decision support framework known as multi-criteria decision analysis (MCDA) has its origins in the field of operations research and has been used in a wide number of fields ranging from environmental management [8,9], agriculture [10], transportation and urban planning [11,12], and to a limited extent in public health [13,14]. In its most basic form, MCDA is a structured tool that allows for the evaluation of alternatives based on multiple, possibly conflicting or even incommensurate criteria in a decision problem. To be more specific, by MCDA we refer only to structured, fully compensatory approaches to decision analysis. A key strength of MCDA is the ability to incorporate multiple stakeholder perspectives as well as uncertain, subjective and qualitative information into an explicit and transparent decision-making process. MCDA based approaches begin with an intelligence phase where the problem definition, decision constraints and evaluation criteria are defined [15]. This is followed by a design phase where the list of possible alternatives and decision-maker's preferences are made explicit [15]. The final phase consists of applying the decision rules and sensitivity analysis in order to produce a recommendation [15]. The general steps involved in the process are described in further detail in Figure 1, and a detailed review of spatial MCDA theory can be found in [15]. Examples of software used in spatial MCDA processes are taken from examples discussed in the review below and listed in Table 1.

**Table 1:** Examples of software and algorithms used in spatial MCDA problem settings

Example decision rules/algorithms	Algorithm strengths	Compatible software packages	Example applications
Analytical Hierarchy Process-specific adaptation of the weighted linear combination method	Multiobjective, multicriteria decision making approach that employs pair-wise comparison procedure to arrive at a scale of preference among a set of alternatives	ArcGIS (ESRI), IDRISI (Clark Labs, Worcester, MA), MultCSynch software package	Aceves-Quesada et al., 2006 [23], Akgun & Turk, 2010 [21], Rakotomanana et al., 2007 [18], Sarkar et al., 2010 [19], Vadrevu et al., 2010 [22]

Compromise programming and spatial compromise programming	Identifies solutions based on their deviations from the ideal solution	ArcGIS (ESRI), MCE-RISK	Chen et al., 2001 [25], Lim & Lee, 2009 [28]

Dempster-Shafer theory	Capable of representing uncertainty based on probability distributions	IDRISI (Clark Labs, Worcester, MA)	Clements et al., 2006 [17]

Fuzzy multicriteria decision-making	Can accommodate non-crisp data	ArcGIS (ESRI)	Chang et al., 2008 [29]

Ordered weighted averages	Provides mechanism to compensate for criteria with low scores via criteria with higher scores	IDRISI (Clark Labs, Worcester, MA)	Clements et al., 2006 [17]

Technique for order preference by similarity to ideal solution	Provides mechanism to compensate for criteria with low scores via other criteria with higher scores	MCE-RISK	Chen et al., 2001 [25]

Weighted linear combination and multicriteria evaluation for weighted linear combination	Fully compensatory model, thought to better represent uncertainty in near-ignorance situations [17]; multicriteria evaluation uses a pairwise comparison method which provides an assessment of the degree of consistency among weightings [31]	IDRISI (Clark Labs, Worcester, MA), MCE-RISK	Akgun et al., 2008 [20], Chen et al., 2001 [25], Clements et al., 2006 [17], Rakotomanana et al., 2007 [18], Symeonakis et al., 2007 [31]

Note: algorithms listed above are generally used in problems of discrete nature. Different algorithms exist for problems with a continuous nature.

The development of these tools has opened up opportunities for extending spatial risk models for vector-borne disease toward a more comprehensive decision support tool. Incorporating new and existing spatial risk models into a MCDA framework would allow for the exploration of alternatives under a wider range of considerations--including the spatial distribution of risk--while providing a credible, recognized and more realistic approach to evidence-based priority setting with enhanced transparency. For example, the spatial arrangement of suitable vector habitat, land use or the distribution of vulnerable populations can be important elements of decisions related to prioritizing vector-borne disease surveillance and intervention strategies. Geographic information systems (GIS), which have already been widely applied to vector-borne disease risk mapping, have been used in conjunction with MCDA, sometimes referred to as GIS-MCDA or spatial MCDA (these terms will be used interchangeably henceforth), to gain insights on the effects of spatial constraints such as zoning, land use or demography on policy-making problems in public health and other disciplines.

Although MCDA has been widely used in a number of disciplines over the last few decades, the use of spatial MCDA remains relatively recent and its application in public health related fields such as vector-borne disease prevention and control is limited. A recent review of the GIS-MCDA literature published between 1990 and 2004 [16] mentions no public health or vector-borne disease-related articles although a few have been written since that time [17-19]. Our objective is to review recent advances in the development of the spatial MCDA-based framework and to illustrate how it can be applied to public health decision support for vector-borne diseases. We aim to demonstrate its broad utility as a decision support tool and illustrate its potential for public health priority setting and control program planning around vector-borne diseases. We propose that this approach has the potential to capitalize on the utility of spatial risk models to evaluate and prioritize targets for prevention and control activities while allowing for the consideration of additional criteria important to decision-makers.

## Review

### MCDA for spatially explicit decision support

Public health problems often have solutions that require decision making, firstly around the assessment of the distribution of risk ("what is the level of risk"), and secondly around risk management which involves deciding between a number of prevention and control strategies ("how to manage the disease risk") or deciding where and/or when to target a given prevention and/or control strategy ("where to target risk prevention and control"). Spatial MCDA has the flexibility to address all of these problem types. We begin by illustrating, with vector-borne disease examples where available, the different categories of decision problems that can be explored using spatial MCDA that target the "what", "how" and "where" questions of disease management. We then propose a conceptual framework to show how these can be used to construct an effective prevention and control program around vector-borne disease management.

#### Risk Assessment: What is the level of risk?

Due to their wide range of environmental determinants, risk for vector-borne diseases is heterogeneously distributed in space, and understanding where risk occurs and how the level of risk can vary between different regions is a frequent subject of study. A commonly encountered use of spatial MCDA involves the assessment of a geographical area based on a perceived threat. Some examples from the environmental sciences include a number of natural hazards such as assessing the risk of forest fires, the risk of volcanic eruptions, and landslide vulnerability [20-25]. One advantage spatial MCDA offers for vector-borne disease risk mapping is the ability to create maps in data-poor environments by incorporating what is already known about the disease, an approach not readily possible with traditional risk mapping techniques. In a study of Rift Valley fever (RVF) in Africa, Clements and colleagues [17] used MCDA to create a knowledge-driven model of disease risk. Based on relationships between environmental drivers and RVF risk, the authors used spatial MCDA to integrate technical spatial information on climate, landscape and livestock density along with data extracted from published literature and expert opinions into suitability maps for both endemic and epidemic RVF activity. Their use of MCDA allowed for the incorporation of what is known about the spatial distribution and uncertainties related to RVF from the published literature in order to create estimates of disease activity at a continent-wide scale. Criticism of their approach includes the subjective nature of parameter estimation and difficulties with validation of the results [26]. However, in the absence of detailed field-based disease surveillance data, one of the key strengths remains an ability to create preliminary estimates of risk that, while imperfect, may still have extensive utility for initial control program planning and priority-setting.

A different approach to using MCDA for risk assessment was shown in a study by Sarkar and colleagues of Chagas disease risk in Texas [19]. In this study, the authors used MCDA to estimate county level risk for Chagas disease by jointly evaluating two risk models, a vector distribution model and a parasite presence model. In this example, spatial MCDA allowed for an understanding of how two representations of potential disease risk interact and offers a method for amalgamating multiple models in order to gain a more robust and complete picture of risk. Using this process, the authors were able to identify areas that may be at risk for Chagas disease even in the absence of reported parasite presence.

#### Selection of alternatives: How to manage disease risk?

The use of MCDA to evaluate possible solutions to a problem, or alternatives, is a common approach seen in environmental and land use decision-making related studies that has been little explored in vector-borne disease contexts. A recent study by Mourits and colleagues [27] explored the use of MCDA to evaluate control strategies against contagious animal diseases but in a non-spatial context. However, many decision problems requiring a selection among a choice of alternatives often involve criteria that are not spatially explicit, but have consequences that are. For example, in a decision context where the primary criteria under evaluation involve cost and other non-spatial criteria, if cost is limiting, then it may not be possible to treat the entire extent of the geographical area at risk. For this, spatial MCDA can be used to evaluate and provide an estimate of the spatial extent of areas affected by different alternatives. For example, in a study by Lim and colleagues [28], spatial MCDA was used to assist in floodplain management in the Suyoung river basin in Korea. In order to select the best strategy from a number of potential alternatives, the authors used compromise programming, the idea behind which is identifying a solution that is as close as possible to an ideal state. In this study, the effect of different stakeholder values on the decision process was evaluated by simulating six different weighting schemes each in turn giving highest weight to one of the evaluation criteria. Resulting maps were produced reflecting each of the weighting schemes in order to visualize the effects of differing values on floodplain management for the study area.

We found no examples of spatial selection of alternatives relating to vector-borne diseases. A hypothetical example of an application of this approach to vector-borne disease management might involve the evaluation of an array of vector control strategies in a particular region in order to determine the best alternative or set of best alternatives among many. For example, given a mosquito-borne disease threat, a list of potential management strategies would be created, which might include a number of pesticide spraying methods or strategies, education about personal protective measures such as the use of insect repellent or bed nets, or larviciding among others. Next, a list of criteria would be developed in order to evaluate the proposed alternatives. One advantage of MCDA is its ability to incorporate diverse and even conflicting values of various stakeholders in a community; therefore, the criteria are selected based on these values as well as any spatial or other constraints relevant to the problem. Criteria could include the reduction of disease-related morbidity and mortality as well as potential adverse effects to human and environmental health from suggested alternatives along with other considerations such as economic costs, duration of effectiveness and efficiency in a given setting. A MCDA approach would allow for the reconciliation of these traditionally incommensurate criteria and would provide an ordering of the proposed alternatives from best to worst given the evaluation criteria specified. Balancing multiple criteria and achieving a thorough understanding of the interaction between criteria and alternatives becomes particularly relevant when the disease in question has a low incidence or is not likely to be fatal, making the impacts of a potentially undesirable intervention such as pesticide use difficult to balance with the protection it provides.

#### Site selection: Where to target risk prevention and control?

Another challenge in public health decision making has to do with determining the best locations for targeting often limited prevention and control resources. MCDA has been used previously to make decisions around "site selection" type problems where decision makers must determine the most suitable locations for a particular facility or activity. In the literature on this subject, the sites under consideration are often intended for noxious activities such as waste management [29] or nuclear power facilities [30], but this approach could be easily extended to decisions around priority sites for deploying a surveillance or vector control strategy. In these problem settings, decision makers must reconcile the choice of location for a particular site that maximizes its efficiency while respecting any related environmental or other concerns.

Site selection can be a contentious issue as decision makers navigate between often times conflicting economic, ecologic and environmental health related constraints in order to make decisions that meet priorities while mitigating potential adverse effects. In addition, there may be a degree of uncertainty related to the parameters under consideration posing a real challenge to any modeled quantification of the problem. Site selection in vector-borne disease management could involve the selection of priority sites to receive control interventions in order to help reduce exposure to a vector and/or pathogen of a vector-borne disease. A study by Symeonakis and colleagues used spatial MCDA for trypanosomiasis management in Zambia [31]. In this study, the authors were interested in prioritizing areas for tsetse fly control (the vector for trypanosomiasis) based on suitable zoning potential and with a high potential to absorb over population and cattle farming expansion from neighboring areas. In order to do this, the authors made use of knowledge on the distribution of tsetse, land designation, bird species richness, cattle density, crop-use intensity and erosion risk data in order to prioritize areas for tsetse control. Of particular interest in this case study was the development of four different hypothetical perspectives during the sensitivity analysis phase in order to simulate the effect of differing values on the decision making process. This is a core strength of an MCDA-based approach as it allows for a transparent understanding of how the values we hold affect our decision making.

Another application of MCDA to a site selection problem was demonstrated by Rakotomanana and colleagues [18] where spatial MCDA was used to evaluate malaria risk in various locations in the highlands of Madagascar in order to make the most effective use of limited vector-control resources and target priority zones to receive indoor spraying against malaria transmitting mosquitoes. A number of spatially-varying risk factors and decision-relevant criteria were incorporated, including altitude, temperature, human population density, time since last indoor spraying, distance from rice fields and surface area of rice fields per district. In this case, the use of GIS-MCDA allowed the incorporation of elements relevant to the decision making process beyond those directly identified by disease-related drivers in order to help guide and assist the selection of priority sites to receive indoor spraying.

#### A unified framework for vector-borne disease risk assessment and risk management

**Figure 2:**
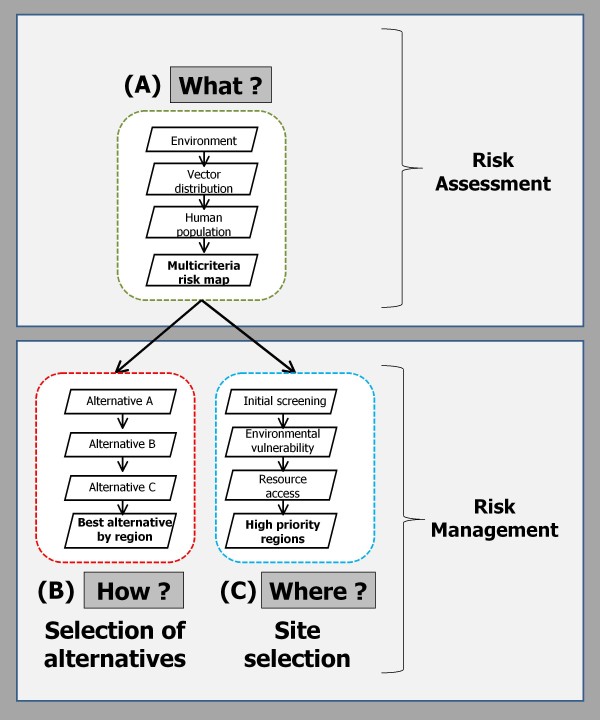
**Key areas for the application of spatial MCDA in managing vector-borne diseases**. Three important questions that require consideration when planning prevention and control actions for the management of vector-borne diseases: what is the level of risk; how to manage disease risk; and where to target risk prevention and control? The above diagram shows how (A) risk can be assessed by mapping the intersection of all the various determinants of risk (environment, vector distribution, human population, etc). Part (B) shows how MCDA can be used to evaluate the spatial effects of different alternatives on a decision problem. Finally, part (C) shows how MCDA can be used to locate priority sites for targeting prevention and control alternatives by taking a risk map and running it through the filter of different criteria constraining the decision problem. Results from one area can be fed in as input for the other questions. However, these three questions do not necessarily need to be addressed in the above suggested order nor will a full MCDA process always be required to address such questions.

As was discussed in the examples above, we saw how MCDA can be used to evaluate questions related to vector-borne disease risk assessment and vector-borne disease risk management. Our review of the literature suggests that the coupling of GIS and MCDA for vector-borne disease management is still relatively unknown; however, the examples explored from other disciplines suggest opportunities to target the "what", "how" and "where" questions that must be considered when constructing effective prevention and control programs for vector-borne diseases. Typically, once a vector-borne or environmental disease threat has been identified, key issues include what the risk of exposure is and what the forecasted burden of disease are to the population; what the best management strategy might be; and where it would be most effective to apply a chosen prevention and control strategy. The use of spatial MCDA provides an opportunity to compare potentially incommensurate data and explore alternative strategies in a spatial context with a potentially unified framework for both risk assessment and risk management based problem settings (Figure 2).

## Conclusions

### MCDA challenges and limitations

The use of MCDA-based methods offers a valuable framework for the exploration of decision alternatives to complex problem settings including ones involving vector-borne disease; however, though many MCDA models exist, no single one will be appropriate for all situations. MCDA-based models are faced with a number of limitations. Perhaps one of the most significant of these is the potential for manipulation of the decision result; however, this is not a challenge unique to these types of models. MCDA models should be used with careful consideration to the sensitivity and robustness of results and should be seen not so much as a decision making tool but rather as a decision aid support. An important caveat when using MCDA to investigate vector-borne disease pertains to causal relationships. In fact, MCDA models are not designed to determine causality but rather to help identify gaps and limits in knowledge of a particular problem setting. MCDA can be used to incorporate data on disease burden; however, if only incorporating data on risk, then the alternatives need to be interpreted with caution as these will not reflect information on actual disease prevalence. MCDA-based models can also be very data intensive both in terms of the material and information required to run the process as well as the data and information necessary to help facilitate stakeholder meetings and are therefore sensitive to inaccuracies or omissions of data [32]. MCDA may also require the participation and engagement of a large number of experts. This later requirement may result in a necessarily lengthy time period for implementation due to the scheduling challenges that inevitably occur when numerous experts and stakeholders are involved [32]. For this reason, MCDA is better suited for long term planning rather than during an emergency/outbreak situation. In addition to this, there is a necessary time lag required for stakeholders to appropriate themselves of the process, or as stated by Bots and Hulshof "grow into the process in order to commit themselves to the process and to the results" [32]. Furthermore, a multi-stakeholder MCDA approach necessarily requires an effective facilitator to keep all stakeholders on track and effectively incorporate contributions of all participants into the process while navigating and synthesizing the input provided by experts and decision makers [32].

### Integration of variable data types and data quality

An important strength provided by a formal MCDA approach is the ability to integrate a wide variety of data types and the ability to accommodate variability in data quality. In their study on RVF, Clements and colleagues [17] showed how knowledge-driven risk assessments can be performed even in the absence of extensive field or digital data. In the study by Sarkar and colleagues we saw how MCDA can be used to combine different types of data, in this case a vector distribution risk model and a pathogen presence model. Symeonakis [31] and Rakotomanana [18] both showed how MCDA can be used to combine traditionally incommensurate forms of information and help prioritize sites for vector control activities. All of these studies illustrate how MCDA can be used to provide an assessment of risk or a prioritization of risk in situations where traditional risk mapping approaches may not be easily performed while providing an opportunity to take the risk modeling process a few steps further in a disease management strategy.

### Flexibility of approach

Risk assessment, selection of alternatives and site selection do not always need to be performed in sequence, and in some disease contexts, the answer to these questions may already be known or constrained by factors which do not necessitate a full and formal MCDA process. We propose that spatial MCDA can be implemented for any or all of these three main questions and can provide a richer picture of constraints on a decision problem as well as an ordered ranking of suitable alternatives to consider given the data available. In their study on floodplain management, Lim showed how spatial MCDA can be used to give a geographical estimation of the effect of different potential flood reduction alternatives in a region of interest [28]. As was seen in the study by Lim and colleagues [28], the selection of alternatives step may not always require a full MCDA process in order to select a suitable alternative depending on the number of alternatives under consideration, or it may be that a non-spatial MCDA process is most appropriate to select among a set of alternatives, but where used, spatial MCDA will enable a visualization of the effects of different proposed alternatives to a decision problem.

### Enrichment of the decision-making process: multiple dimensions and multiple stakeholders

Additionally, the use of a formal MCDA approach for problem solving enriches the decision-making process by making explicit the multiple dimensions of a problem as well as the values held by stakeholders. In the study by Symeonakis and colleagues [31] we saw how MCDA can help illuminate the effect of differing values on the decision making process. Although multi-stakeholder perspectives were not predominantly explored in the vector-borne disease studies we reviewed, we wish to emphasize that the MCDA process is designed to accommodate multiple stakeholders and this approach has been well field tested in many of the other disciplines that have a longer history of MCDA use. This particular feature of MCDA provides an important opportunity for public health policy-making to incorporate diverse viewpoints in decision making and is one that should be further explored in future studies.

### Formal and transparent interpretation of results

Public health policy related to the prevention and control of vector-borne disease requires the consideration of diverse and often difficult to estimate criteria. In addition to disease risk, which may itself be a challenge that can benefit from a multi-criteria approach, policy-makers must consider a range of other constraints. Many interventions for vector-borne diseases such as pesticide use or vector habitat modification have potentially harmful effects on the environment and on wildlife populations that should be weighed against their benefits. Ecological approaches to controlling vector-borne diseases can be associated with high personnel and equipment costs and are often variably effective depending on the setting. The challenges of preventing and controlling vector-borne diseases have few obvious answers. The strengths of this approach lie not only in its ability to help reconcile conflicting values and find a consensus decision among differing perspectives, but also in its utility as a tool for understanding the issues at stake and inherent to the decision process itself. MCDA is an interesting tool capable of capturing the complexity and inherent multidisciplinarity of vector-borne disease contexts and other diseases at the human-environment interface, and thus is an invaluable tool for exploring and facilitating decision-making in these contexts.

## List of abbreviations used

GIS: geographical information systems; MCDA: Multi-criteria decision analysis; RVF: Rift Valley fever.

## Competing interests

The authors declare that they have no competing interests.

## Authors' contributions

VH and AGH contributed to conception, design and analysis and drafted the manuscript. CA and PM contributed to conception and analysis as well as critical revision of the manuscript. JPW, DB and LMC contributed to the critical revision of the manuscript. All authors read and approved the final manuscript.
